# Genotypic and antimicrobial resistance characterizations of *Cronobacter sakazakii* isolated from powdered milk infant formula: A comparison between domestic and imported products

**DOI:** 10.1002/fsn3.1965

**Published:** 2020-10-30

**Authors:** Babak Pakbin, Razzagh Mahmoudi, Shaghayegh Mousavi, Samaneh Allahyari, Zahra Amani, Amir Peymani, Peyman Qajarbeygi, Zahra Hoseinabadi

**Affiliations:** ^1^ Department of Food Hygiene and Quality of Control Faculty of Veterinary Medicine University of Tehran Tehran Iran; ^2^ Medical Microbiology Research Center Qazvin University of Medical Sciences Qazvin Iran; ^3^ Faculty of Medical Sciences Department of Molecular Medicine Qazvin University of Medical Sciences Qazvin Iran; ^4^ Department of Food Hygiene and Safety School of Health Qazvin University of Medical sciences Qazvin Iran; ^5^ Health Products Safety Research Center Qazvin University of Medical sciences Qazvin Iran

**Keywords:** antimicrobial susceptibility, *Cronobacter sakazakii*, genotyping, powdered milk infant formula

## Abstract

*Cronobacter sakazakii*, an opportunistic foodborne pathogen and a main cause of meningitis in neonates, is usually isolated from powdered milk infant formula (PMIF). At the present study, *C. sakazakii* were isolated from imported and domestically produced PMIF samples and identified by detection of *ompA* gene using real‐time PCR SYBR green melting curve following the evaluation of antimicrobial susceptibility and genotyping of the isolates employing BOX‐PCR and RAPD methods. We detected totally 5% contamination rate and a significantly higher prevalence of *C. sakazakii* in bulky imported domestically packaged PMIF samples. Also, our isolates were recognized as multidrug‐resistant pathogen completely resistant to ampicillin and amoxicillin; and intermediately resistant to ciprofloxacin and tetracycline antimicrobials. Genotype clustering patterns of bulky imported and imported product isolates were identical by both genotyping methods. Far genetic relatedness of domestic isolate to other isolates and the reference strain indicated higher genetic diversity of the domestic isolate genome. Multidrug resistance and diverse population genetic make complicated situation for determination of strategies for infectious disease prevention.

## Graphical abstract

*Cronobacter sakazakii* strains were isolated from imported and domestically produced PMIF samples and identified by detection of ompA genes using real‐time PCR SYBR green melting curve following the evaluation of antimicrobial susceptibility and genotyping of the isolates employing BOX‐PCR and RAPD methods. A close relationship between genotype clustering and antimicrobial susceptibility patterns showed that drug resistance properties of *C. sakazakii* isolates affect genetic diversity and genomic evolution.

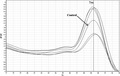

## 1 INTRODUCTION

Not appreciated by pediatric and nutrition specialists, powdered milk infant formula (PMIF) is the most recommended alternative of breast milk for neonatal feeding (Lönnerdal, 2016). Several types of research appreciated the importance of safety and microbial quality for powdered milk and its derivatives consumed by infants and newborns (Martin, Ling, & Blackburn, 2016). Milk, before drying, is a very nutritive and suitable growth medium for a wide range of disease‐causing and spoilage bacteria. However, after drum or spray drying and during packaging many foodborne pathogens, for example, *Salmonella* spp., *Cronobacter* spp., and *Escherichia coli*, contaminate the powdered product and survive for a long time (Losio et al., 2018). Some of these pathogens seriously threaten the health of consumers and cause to lethal diseases, for example, meningitis, bloody diarrhea, and sepsis in newborns and infants. Several studies recognized the importance of monitoring and surveillance of PMIF regarding detection and identification of some prevalent pathogens (Yang et al., 2014).

*Cronobacter sakazakii*, an emerging opportunistic foodborne pathogen known worldwide and belonging to the *Enterobacteriaceae* family, cause many diseases in adults and infants including meningitis, sepsis, necrotizing enterocolitis, and acute diarrhea (Henry, 2018). This pathogen is one of the main causes of life‐threatening agents in PMIF, and some powdered and dried products recently recognized as the indicator of safety performance by many researchers and food safety organizations. A mortality rate range between 40% and 80% for *C. sakazakii* causing meningitis and septicemia in infants and newborns was recently reported (Zeng et al., 2018). This pathogen has been detected in a wide range of food items consisting of milk, meat, cheese, grains, vegetables, spices, and herbs; however, PMIF is the most sensitive one (Aly et al., 2019). It has been detected and identified by many rapid, sensitive, and specific assays, as well as molecular, immunological, and biosensor techniques in PMIF samples (Kim et al., 2017). One of these successful methods previously employed for identification of *C. sakazakii* in PMIF samples is real‐time PCR SYBR green melting curve assay using species‐specific primers. Considering the precise and specificity of melting temperature analysis of PCR amplicons rather than evaluation by gel electrophoresis, melting curve analysis of PCR products is more precise, specific, and sensitive than conventional PCR (Cai et al., 2013). There are many genes employed as species‐specific markers of *C. sakazakii* for identification of this pathogen, for example, *ompA*, *gluA*, *zpx*, *sodA*, and *gyrB* (Singh, Goel, & Raghav, 2015). *OmpA* gene encodes outer membrane proteins A of *C. sakazakii* contributing to invasion of human brain endothelial cells considering the main virulence factor of this pathogen causing meningitis in newborn babies; consequently, it has been used as the marker of identification of *C. sakazakii* by researchers (Mohan Nair, Venkitanarayanan, Silbart, & Kim, 2009).

Antimicrobial‐resistant foodborne pathogens which have more been investigated in recent decades are a major public health concern. Exposure to antimicrobial treatment in clinical practices and animal production makes selective pressures to emergence of antimicrobial resistance phenotypic properties and genes in pathogens (Blair et al., 2015). Antimicrobial resistance properties increase the mortality and morbidity of infectious diseases caused by the pathogen and contribute to a huge socioeconomic cost. Increasing trend of antimicrobial resistance in foodborne pathogens makes antimicrobial treatments insufficient in the future contributing to emergence of new generations of pathogens thereby the public health concern will be raised drastically. Consequently, surveillance and monitoring of antimicrobial patterns of foodborne pathogens are extremely essential to be investigated frequently (Frieri et al., 2017). Also, antimicrobial‐resistant *C. sakazakii* isolates caused meningitis and sepsis infections have raised concerns about the treatment of associated infectious diseases. Multidrug resistance properties are developed in *C. sakazakii* isolated from PMIF because of the evolution as well as selective pressures and exposure of this pathogen to environments containing different classes of antimicrobials (Parra‐Flores, Aguirre, et al., 2018).

Several methods may be employed to investigate genetic diversity of bacteria for tracing and classification of pathogens in outbreaks and surveillance studies (Li et al., 2009). Pulsed field gel electrophoresis (PFGE), BOX‐polymerase chain reaction (BOX‐PCR), repetitive sequence‐based PCR (rep‐PCR), multilocus sequence typing (MLST), random amplification of polymorphic DNA (RAPD), enterobacterial repetitive intergenic consensus PCR (ERIC‐PCR), and ribotyping methods have been employed previously for genotyping of *C. sakazakii* isolated from PMIF samples (Xu et al., 2015). Several studies found BOX‐PCR and RAPD methods suitable, precise, and low cost for fingerprint genotyping, phylogenetic tree analysis, and genotype categorization of *C. sakazakii* isolates (Kakatkar et al., 2017). The aim of this study was to investigate the antimicrobial susceptibility, evaluation and genotyping of *C. sakazakii* isolated from imported and domestically produced PMIF samples.

## 2 MATERIALS AND METHODS

### 2.1 Sample collection

Totally 118 infant formula samples have been collected including different brands from pharmacies located in different areas of Qazvin, Iran, from February to July 2018. These samples were classified in three sample types including 24 imported (from United Kingdom, Spain, Netherlands, and Germany) products (I); 50 bulky imported (from different countries consisting of Belgium, Germany, and Netherlands) and domestic products (B); and 44 domestic products (D) which were formulated and packaged by different producers in Iran. All samples were transported and stored in original package form before the primary microbial isolation step for *C. sakazakii* detection.

### 2.2 Isolation of *C. sakazakii*

**FIGURE 1:**
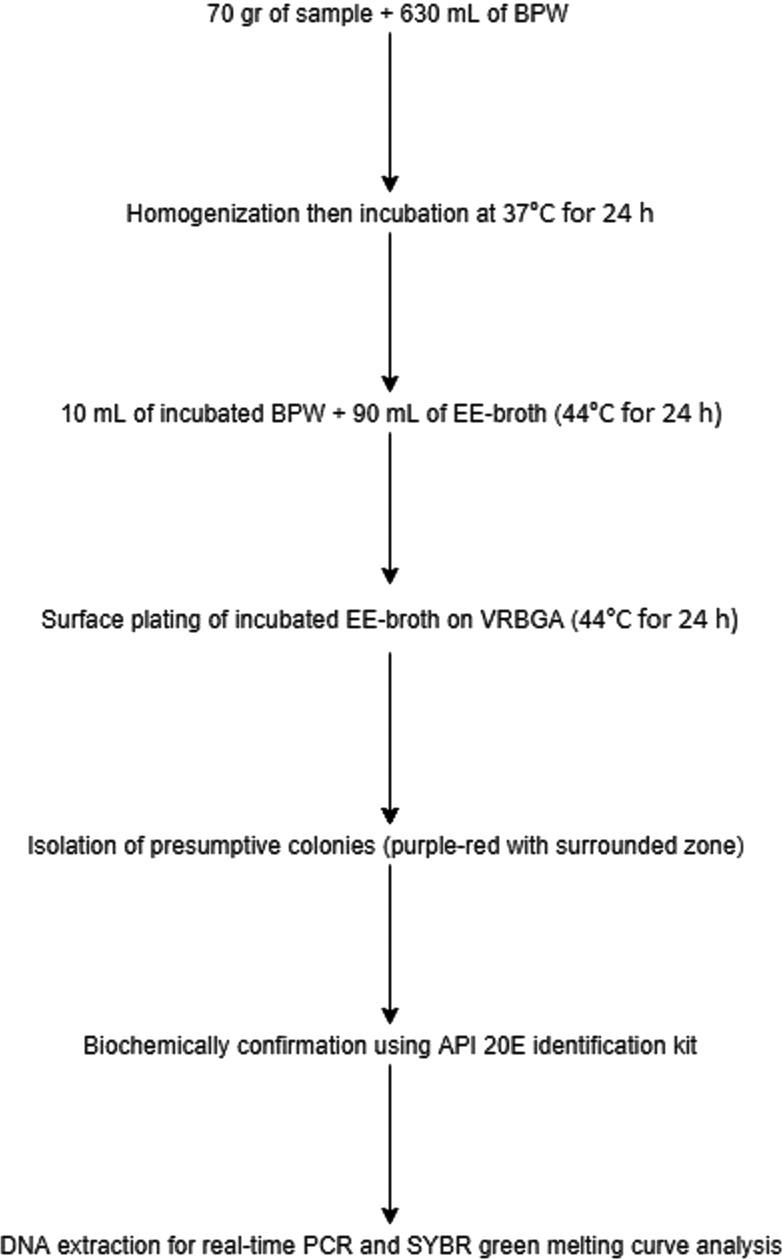
Flowchart of culture‐based method for isolation of presumptive *C. sakazakii* from PMIF samples

Primary isolation and presumptive identification of *C. sakazakii* was carried out based on the method described by *Microbiological examination methods of food and water: a laboratory manual* (Da Silva et al., 2018) as shown in Figure 1. For each sample, after opening the original PMIF package under aseptic condition, seventy grams of homogenized PMIF was dissolved, diluted, and pre‐enriched selectively with 630 ml of sterilized buffered peptone water (BPW, ProMedia) following incubation at 37°C for 24 hr. For enrichment purpose, 10 ml of incubated BPW culture was mixed with 90 ml of Enterobacteriaceae Enrichment broth media (EE‐broth, ProMedia) at 44°C for over a 24‐hr period. *C. sakazakii* was isolated and presumptively detected from the enrichment media by surface plating on Violet Red Bile Glucose Agar (VRBGA, ProMedia) for 24 hr at 44°C. The colonies with purple‐red and same color surrounded zone formation were subjected to API 20E identification system (BioMerieux) for biochemical confirmation and then considered as presumptive *C. sakazakii* isolates for molecular identification in the next steps. *Cronobacter sakazakii* ATCC 29,544, as standard and positive control strain, was grown in tryptic soy broth (TSB, ProMedia) for 24 hr at 37°C.

### 2.3 DNA extraction

For genomic DNA extraction of presumptive *C. sakazakii* isolates, single colonies were picked up from VRBGA isolation medium for inoculation into 3 ml of Brain Heart Infusion broth (BHI‐broth, ProMedia) for overnight at 37°C with 200 rpm shaking. After the incubation, flasks were centrifuged at 5,000 × *g* for 10 min; the biomass pastes were used for DNA extraction after removing the supernatant. The DNA extraction procedure was carried out using Cinnagen commercial gram‐negative bacterial DNA extraction kit (Cinnagen Co. Iran) according to the manufacturer instruction. The purity and quantity of the extracted genome were measured by NanoDrop Spectrophotometer (Thermo Fisher Scientific Co.), and the DNA samples were stored at −20°C until the further investigations.

### 2.4 Identification of *C. sakazakii* by real‐time PCR SYBR green melting curve

Real‐time PCR method was used for identification and confirmation of *C. sakazakii* at the present study. Species‐specific primers of *ompA* gene, presenting in *C. sakazakii,* used for the PCR are described in Table 1 (Kilonzo‐Nthenge et al., 2012), and they were synthesized by the Cinnagen company (Cinnagen Co. Iran). The real‐time PCR mix contained 10 µl of 2X SYBR green real‐time PCR master mix (Ampliqon), 3 µl of DNA templates (50 ng/µl), 1.5 µl of each primer (10 µM) and addition of deionized sterile water to the final reaction volume (20 µl). A Rotor‐Gene 6,000 real‐time PCR machine (QIAGEN) was employed for thermal cycling and recording of fluorescence changes. The PCR was performed as follows: 2 min at 95°C for initial denaturation and then 30 cycles of 15 s at 95°C, 15 s at 60°C, 30 s at 72°C as denaturation, annealing, and elongation steps, respectively. Melting curve analysis of the PCR products was performed at temperatures between 60 and 95°C with the raising temperature rate of 0.2°C/s. Melting curves were obtained and analyzed by the Rotor‐Gene 6,000 software version 1.7 (QIAGEN) associated with the real‐time PCR machine.

**Table 1:** Primer sequences used for real‐time PCR assay and genotyping methods

Primer	GenBank no.	Sequence	Amplicon size (bp)	References
ompA‐F	DQ000206	3′‐GGATTTAACCGTGAACTTTTCC‐5′	469	Kilonzo‐Nthenge et al. 2012
ompA‐R		5′‐CGCCAGCGATGTTAGAAGA‐3′		
BOX‐PCR	–	5′‐CTACGGCAAGGCGACGCTGACG‐3′	–	Proudy et al. 2008
RAPD	–	5′‐CGCGTGCCAG‐3′	–	Ye et al., 2010

### 2.5 Antimicrobial susceptibility testing

Antimicrobial susceptibility of the confirmed isolates was carried out using disk diffusion method using Mueller‐Hinton agar (MHA, ProMedia, Spain). Eight antibiogram disks were employed (ROSCO Co. Denmark): tetracycline (30 µg), levofloxacin (5 µg), ampicillin (10 µg), amikacin (30 µg), amoxicillin (30 µg), chloramphenicol (30 µg), imipenem (10 µg), ciprofloxacin (5 µg), and cefepime (30 µg). The susceptibility profile of the isolates was evaluated by measuring diameters of inhibition areas and interpreted according to the CLSI guidelines. Escherichia coli ATCC 25,922 and Staphylococcus aureus ATCC 25,923 were used for test quality control as reference organisms. All results were expressed as resistant (R), intermediate (I), and sensitive (S) and interpreted based on CLSI method (CLSI, 2016).

### 2.6 Molecular fingerprint genotyping methods

#### 2.6.1 BOX‐PCR

Repetitive based typing with BOX‐PCR was carried out by the primer described in Table 1 (Proudy et al., 2008). BOX‐PCRs were performed in 50 µl volume reaction containing 25 µl 2X PCR master mix (Ampliqon, Denmark), 2 µl primer (0.5 µM/ml), 2 µl of DNA template (50 ng/µl), and sterilized nucleic acid free water to the final volume. Thermal cycling process was performed with a thermal cycler (Eppendorf) as follows: initial denaturation at 95°C for 5 min then 30 cycles including 30 s at 95°C and 1 min at 55°C, finally 8 min at 72°C as final extension step. PCR products were separated by gel electrophoresis on 1.5% agarose gel (Merck) with 0.005% v/v red safe staining dye (Ampliqon) and characterized by a 100‐bp standard marker (Ampliqon, Denmark). Photograph of the gel was taken by charge‐coupled device (CCD) camera, and the fingerprint was visually interpreted by PyElph software version 1.4 (Pavel & Vasile, 2012) following calculation of similarities and drawing of unweighted pair group method with arithmetic mean (UPGMA) dendrogram by NTsys software version 2.1 (Rohlf, 1999).

#### 2.6.2 RAPD

The primer used for RAPD genotyping of the isolates at the present study is presented in Table 1 (Ye et al., 2010). The PCR mixture (25 µl) consisted of 12.5 2X PCR master mix, 1 µl of primer (0.2 µM/ml), 2 µl of DNA template (50 ng/µl), and deionized sterile water bringing the reaction volume to 25 µl. The RAPD was carried out using thermal cycling program as follows: 95°C for 5 min, then 1 min at 36°C and 4 min at 72°C followed by 35 cycles of 1 min at 95°C, 1 min at 36°C and 72°C for 4 min. The PCR products were characterized by 1.5% agarose gel electrophoresis at 100 V for 1.5 hr; finally, the photograph of the gel was captured by CCD camera under UV transilluminator. Similar to previous genotyping methods, all analysis and drawing UPGMA dendrogram were implemented using PyElph and NTsys softwares.

### 2.7 Statistical analysis

Fisher's exact and chi‐square tests were employed for evaluation of significant differences (*p* < .05) between contamination rates using SPSS software version 22.0.1. Also, all experimental and statistical measurements were implemented in triplicate.

## 3 RESULTS

### 3.1 Detection and identification of *C. sakazakii*

**FIGURE 2:**
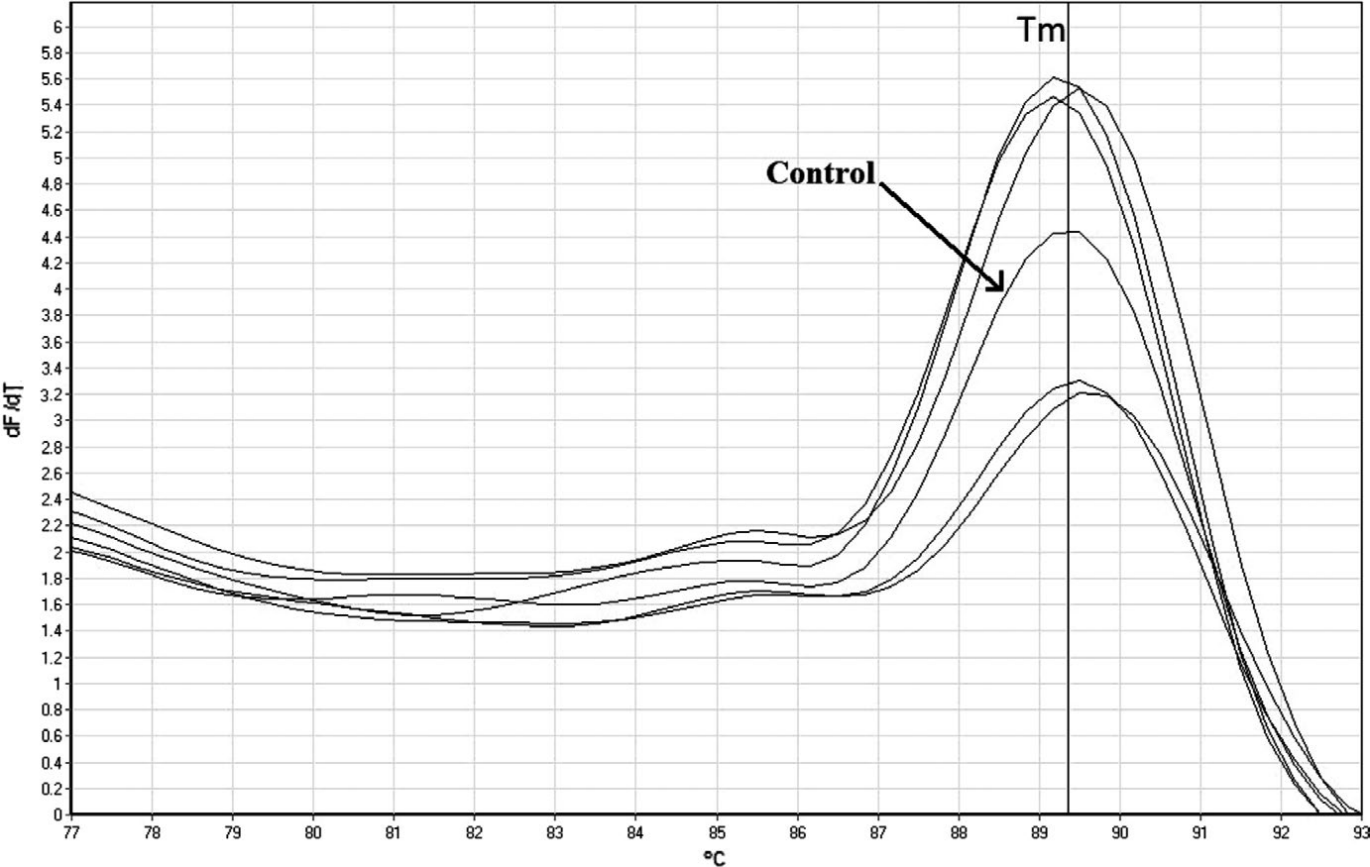
Melting temperature of PCR products for identification of ompA gene among presumptive *C. sakazakii* isolates, including positive and control samples (Tm = 89.4°C)

**FIGURE 3:**
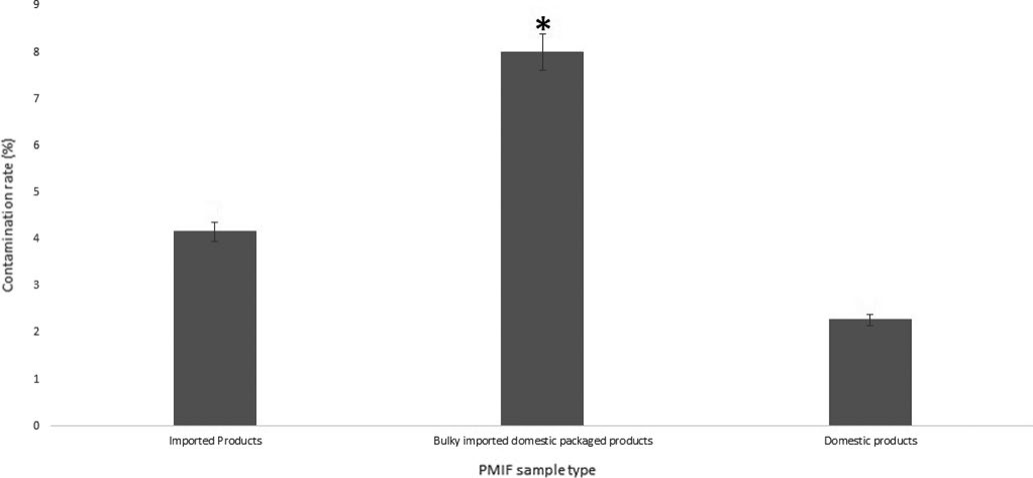
Contamination rate of *C. sakazakii* between different PMIF sample types. * indicates significant differences (*p* < .05) based on Fisher's exact test between the contamination rates of *C. sakazakii* between the different sample types

At the present study, *C. sakazakii* was isolated and identified from PMIF samples by culture‐based and real‐time PCR SYBR green melting curve methods, respectively. PMIF samples collected from different pharmacies located in Qazvin, Iran, in the basis of three‐type categorization consisting of imported, bulky imported and domestic packaged; and completely domestic PMIF products. After culture‐based detection and biochemically confirmation (by API 20E kit), presumptive *C. sakazakii* was isolated in 10 PMIF packages totally among all samples. Employing identification with real‐time PCR SYBR green melting curve assay, finally presence of *C. sakazakii* was confirmed in totally 6 (5%) PMIF samples using species‐specific primers for detection of *ompA* gene which indicates there were 4 false positive results detected by the culture‐based identification procedure or maybe 4 isolates did not harbor the *ompA* gene; however, regarding ompA is a housekeeping gene, we chose it because it is one of the main factors of virulence factor encoded gene in *C. sakazakii*. Concentration and 260/280 ratio of the extracted DNA were observed 56 µg/ml and 1.86, respectively, which indicate appropriate quantity and purity properties of the DNA extraction procedure. Real‐time PCR assay for identification of *C. sakazakii* was optimized at the present study for sensitive and specific detection and identification of this pathogen in PMIF samples. DNA templates extracted from *C. sakazakii* ATCC 29,544 strain and deionized water were used as positive and negative samples, respectively. Melting curves of positive samples and the positive control template are shown in Figure 2 with the melting temperature 89.4°C. Because the procedure was optimized for this study, there is not any unspecific melting temperature (amplified product) and positive fluorescence signal from negative controls. As can be seen in Figure 3, the prevalence rates between different PMIF sample type consisting of imported, bulky imported domestic packaged, and complete domestic products were determined 4.16, 8, and 2.27%, respectively. It should be pointed out that the contamination rate of *C. sakazakii* was observed significantly (*p* < .05) higher in bulky imported domestic packaged product type samples than that in other types.

### 3.2 Antimicrobial susceptibility of the isolates

For phenotypic antimicrobial susceptibility evaluation, nine antimicrobials were tested on 6 molecularly confirmed *C. sakazakii* isolates. Two samples (S251 and S361 both are bulky imported type) were detected as the most resistant and sensitive isolates respectively. Isolates from domestic and imported PMIF products showed higher resistance to ampicillin, amoxicillin, and chloramphenicol. However, isolated from bulky imported products detected more susceptible to chloramphenicol, levofloxacin, and amikacin. The results showed strong associations between antimicrobial susceptibility properties of the isolates including complete resistance to ampicillin and amoxicillin; intermediate sensitivity to ciprofloxacin and tetracycline; and complete sensitivity to amikacin and levofloxacin.

### 3.3 Genotyping

**FIGURE 4:**
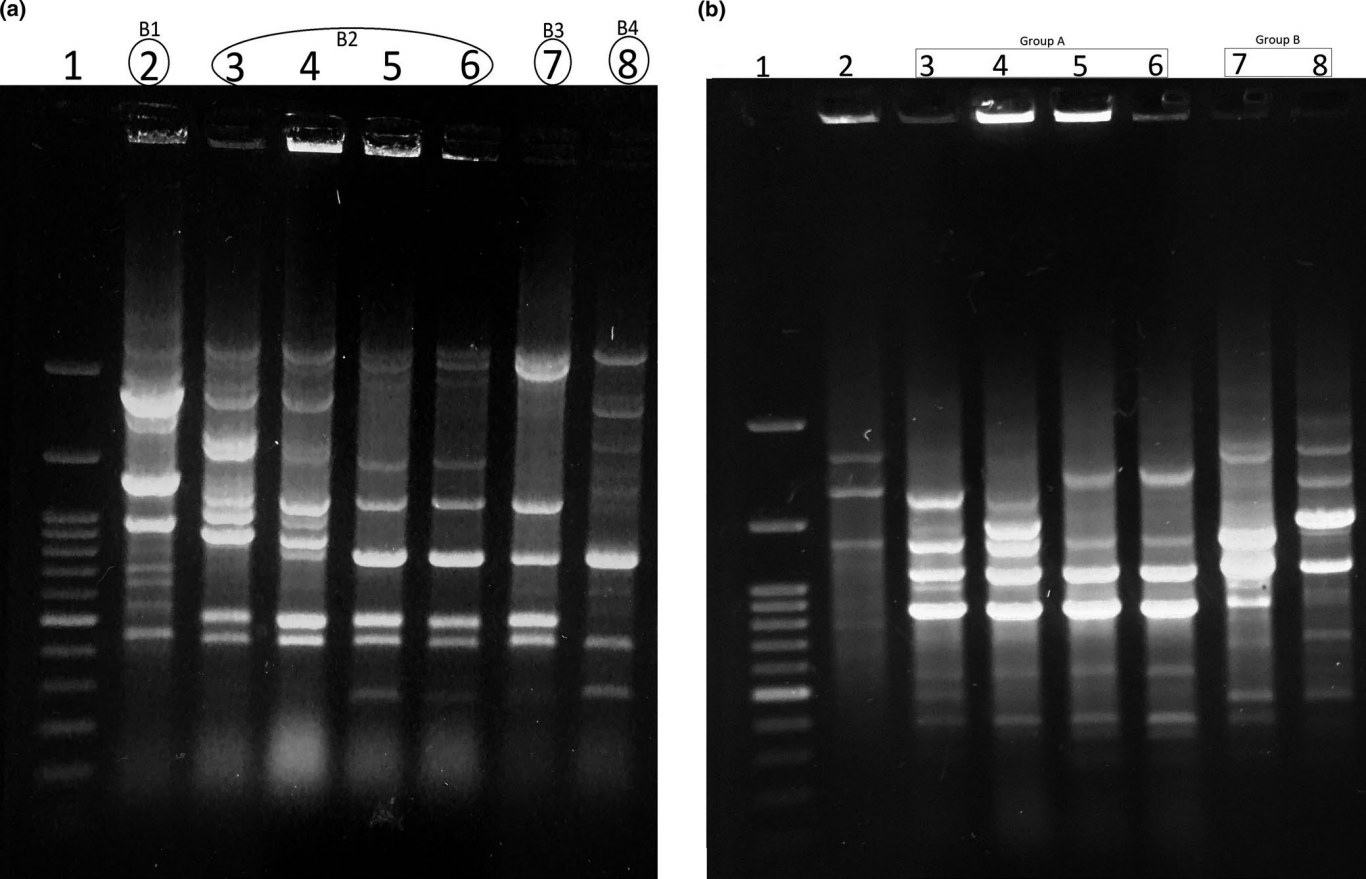
BOX (A) and RAPD (B) results of *C. sakazakii* strains isolated from powdered milk samples (lane 1:100‐bp DNA ladder and lane 2: *C. sakazakii* ATCC 29,544 in both figures a and b; lanes 3–8 in the figure a as S210I, S251B, S341B, S211B, S361B, and S391D and lanes 3–8 in the figure b as S361B, S391D, S210I, S251B, S341B, and S211B)

**FIGURE 5:**
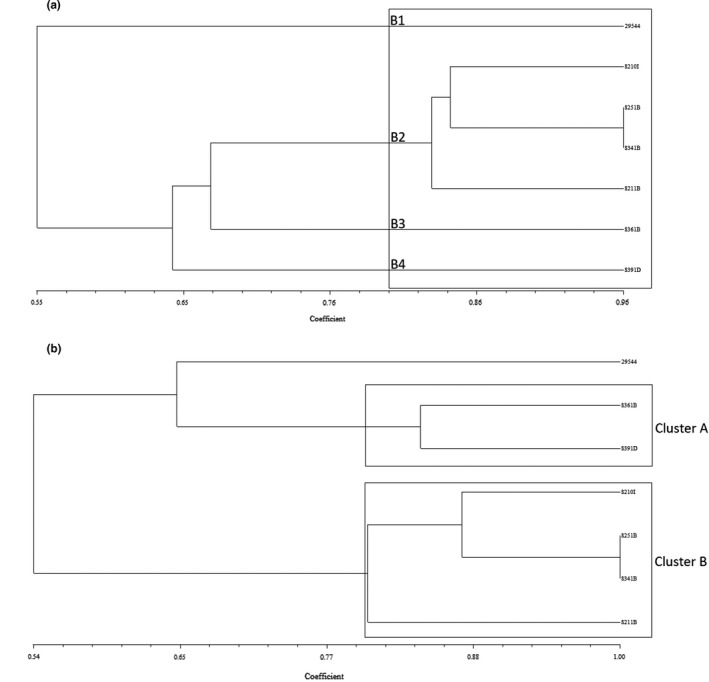
UPGMA dendrograms with the Dice coefficient of *C. sakazakii* isolates in the basis of BOX (A) and RAPD (B) patterns (29,544 as the ATCC reference strain; S210I as imported sample; S251B, S341B, S361, and S211B as bulky imported samples; and S391D as domestic product)

Figures 4a and 5a present the results and dendrogram of BOX‐PCR analysis for *C. sakazakii* ATCC 29,544 and the six isolates consisting of S210I as imported sample; S251B, S341B, S361, and S211B as bulky imported samples; and S391D as domestic product sample. The BOX‐PCR fingerprints consisted of eleven main bands with the ranging size between 200 and 2000 bp. BOX‐PCR patterns resulted different UPGMA clusters in which the isolates clustered distinguishably in the same group were closely genetically related and the unrelated isolates were differentiated into the separate clusters. Four clusters were detected through the dendrogram of BOX‐PCR with the Dice coefficient higher than 80% (B1‐B4). Cluster B2 consisted of four isolates (3 isolates of bulky and one isolate of imported type) closely genetically related to each together. The control isolate (29,544) was detected genetically far from the domestic isolate by BOX‐PCR genotyping. Isolate from imported sample was genetically related to bulky imported type ones and genetically far from the domestic isolate indicating different population genetics of *C. sakazakii* isolated from PMIF. RAPD primer indicated 4–12 bands with the range size of 0.3–2.0 kbp (Figure 4b). This method revealed two clusters (A and B) which are shown in Figure 5b with a Dice coefficient higher than 80%. Three samples (S211, S251, and S341 as bulky imported type) and one sample (S210 as imported product type) were included in a genomic cluster (B). Cluster A contained domestic and another bulky imported sample identified genetically unrelated to the other samples and the control strain ATCC 29,544. Control strain is genetically different from the other samples regardless of the product type and categorized into an individual cluster. Except clustering of domestic isolates, fingerprint genotyping patterns obtained by RAPD and BOX‐PCR methods for *C. sakazakii* isolates were identical.

## 4 DISCUSSION

At the present study, we found 5% of the PMIF samples contaminated with *C. sakazakii* harboring the *ompA* gene which encodes the invasion mechanism in meningitis pathogenesis and has the protective role against the defense mechanism of the host (Holý et al., 2019). We identified *C. sakazakii* in PMIF samples by real‐time PCR melting curve assay in our study; however, there are many researchers suggest SYBR green melting curve real‐time PCR with species‐specific primers a precise, sensitive and specific method for detection and identification of *C. sakazakii* in food samples as well as PMIF (Mashoufi et al., 2019). Recently; because of the employing strong surveillance and checking quality control of the manufactured and imported PMIF products, prevalence of *C. sakazakii* in PMIF has significantly been decreased (Lu & Matthews, 2019). However, different range of prevalence from 2.8% to 12.3% contamination rates of *C. sakazakii* was reported by researchers about PMIF samples (Henry, 2018). Our study contributed toward to a deep understanding and improved surveillance of *C. sakazakii* isolated from commercial imported and domestically produced PMIF. We detected higher contamination rate of *C. sakazakii* significantly according to the Fisher's exact test in bulky imported type samples. Lower hygienic conditions of powder packaging process is the main cause of the observed higher prevalence (Parra‐Flores, Cerda‐Leal, et al., 2018). Also, contaminated spray drier nozzle is considered as a major cross contamination causes leads to prevalence of *C. sakazakii* in domestically produced PMIF; however, this opportunistic foodborne pathogen can survive in dry environments as well as infant formula for a long time (Saad & Ewida, 2018). Because of the lower prevalence in domestic type samples at the present study, it is concluded that the main problem of *C. sakazakii* prevalent in PMIF in Iran is the poor hygienic condition in powder packaging process of bulky imported PMIF.

We found that the 6 *C. sakazakii* isolates were multidrug‐resistant pathogens. They were resistant to ampicillin, amoxicillin, tetracycline, and ciprofloxacin which are supported by previous studies about the isolates from PMIF (Chen et al., 2019; Parra‐Flores, Aguirre, et al., 2018). The domestic isolate was completely and intermediately resistant to all antimicrobials tested at the present study indicating the emergence of concern about the prevalence of antimicrobial‐resistant *C. sakzakii* in PMIF and a serious public health problem. Li et al. (2016) detected *C. sakazakii* isolates in milk‐based foods as multidrug‐resistant foodborne pathogens which were completely resistant to ampicillin, amoxicillin, ciprofloxacin, and some other antimicrobials intermediately (Li et al., 2016). However, some studies revealed that *C. sakazakii* isolated from infant formula were completely resistant to ampicillin and amoxicillin but susceptible to ciprofloxacin and tetracycline (Fei, Jiang, Feng, et al., 2017). Different antimicrobial resistance patterns between domestic and imported PMIF isolates revealed that antimicrobial consumption and exposure by which infection treatment and animal production employ significantly affect the resistance behavior of local isolates as some studies described this phenomenon previously (Fei, Jiang, Jiang, et al., 2017).

Genotyping methods help to better understand and trace dissemination and persistence of *C. sakazakii* in the manufacturing and distribution processes of PMIF as well as for imported or domestic products which it is investigated at the present study. We implemented BOX‐PCR and RAPD assays for genotyping *C. sakazakii* isolates. All isolates of bulky imported (except one isolate) and imported product types were clustered in one group by both genotyping methods in this study revealed that domestic *C. sakazakii* isolates are genotypically different from isolates of imported PMIF products. Also, all isolates were different from the control strain (ATCC 29,544) indicates that *C. sakazakii* isolates have been evolved because of exposure to some selective pressures recently regardless type of the product from which the pathogen was isolated (Fei et al., 2018). Close related clustering and fingerprint genotyping analysis showed that this genetic diversity may be originated from harboring antimicrobial resistance genes which have been located into the genome of the isolates during the evolution (Li et al., 2017). Nevertheless, this hypothesis needs to be investigated more for better clarifying the dynamic genome aspects of the evolution occurred in *C. sakazakii* isolates employing high throughput methods, for example, next generation sequencing and omics technologies (Aly et al., 2019; Zeng et al., 2017).

## 5 CONCLUSIONS

In our study, *C. sakazakii* isolated from imported and domestically produced PMIF samples and identified by detection of *ompA* gene using real‐time PCR SYBR green melting curve analysis with species‐specific primers. After confirmation of *C. sakazakii*, antimicrobial susceptibility testing and genotyping of the isolates were implemented. Higher prevalence of *C. sakazakii* isolates was detected in bulky imported product type; also, totally 5% contamination rate was observed. We found that the 6 *C. sakazakii* isolates multidrug‐resistant at the present study completely resistant to ampicillin and amoxicillin. Low genetic diversity was observed among the isolates; however, far genetic relatedness was detected between the domestic isolate and the reference strain. Close genetical relatedness and genotype clustering indicate that same evolutionary selective pressure forms genetic diversity in our isolates. However, complement studies employing high throughput technologies are needed to be implemented for more precise investigation of these relationships and hypothesis.

## CONFLICT OF INTEREST

All authors declare that they have no conflict of interest.

## References

[fsn31965-bib-0001] Aly, M. A. , Domig, K. J. , Kneifel, W. , & Reimhult, E. (2019). Whole genome sequencing‐based comparison of food isolates of *Cronobacter sakazakii* . Frontiers in Microbiology, 10, 1464 10.3389/fmicb.2019.01464 31333604 PMC6615433

[fsn31965-bib-0002] Blair, J. M. , Webber, M. A. , Baylay, A. J. , Ogbolu, D. O. , & Piddock, L. J. (2015). Molecular mechanisms of antimicrobial resistance. Nature Reviews Microbiology, 13(1), 42–51. 25435309 10.1038/nrmicro3380

[fsn31965-bib-0003] Cai, X.‐Q. , Yu, H.‐Q. , Ruan, Z.‐X. , Yang, L.‐L. , Bai, J.‐S. , Qiu, D.‐Y. , & Le, T. H. (2013). Rapid detection and simultaneous genotyping of Cronobacter spp. (formerly Enterobacter sakazakii) in powdered infant formula using real‐time PCR and high resolution melting (HRM) analysis. PLoS ONE, 8(6), e67082. 23825624 10.1371/journal.pone.0067082 PMC3692429

[fsn31965-bib-0004] Chen, Y. , Zhang, Y. , Wang, X. , Ling, J. , He, G. , & Shen, L. (2019). Antibacterial activity and its mechanisms of a recombinant Funme peptide against Cronobacter sakazakii in powdered infant formula. Food Research International, 116, 258–265. 10.1016/j.foodres.2018.08.030 30716944

[fsn31965-bib-0005] CLSI (2016). Performance standards for antimicrobial susceptibility testing. Clinical Lab Standards Institute, 35(3), 16–38.

[fsn31965-bib-0006] Da Silva, N. , Taniwaki, M. H. , Junqueira, V. C. , Silveira, N. , Okazaki, M. M. , & Gomes, R. A. R. (2018). Microbiological examination methods of food and water: A laboratory manual. CRC Press.

[fsn31965-bib-0007] Fei, P. , Jiang, Y. , Feng, J. , Forsythe, S. J. , Li, R. , Zhou, Y. , & Man, C. (2017). Antimicrobial and desiccation resistance of Cronobacter sakazakii and C. malonaticus isolates from powdered infant formula and processing environments. Frontiers in Microbiology, 8, 316. 28303125 10.3389/fmicb.2017.00316 PMC5332417

[fsn31965-bib-0008] Fei, P. , Jiang, Y. , Gong, S. , Li, R. , Jiang, Y. , Yuan, X. , & Ali, M. A. (2018). Occurrence, genotyping, and antimicrobial susceptibility of cronobacter spp. in drinking Water and Food Samples from Northeast China. Journal of Food Protection, 81(3), 456–460. 10.4315/0362-028X.JFP-17-326 29474142

[fsn31965-bib-0009] Fei, P. , Jiang, Y. , Jiang, Y. , Yuan, X. , Yang, T. , Chen, J. , & Forsythe, S. J. (2017). Prevalence, molecular characterization, and antimicrobial susceptibility of *Cronobacter sakazakii* isolates from powdered infant formula collected from Chinese retail markets. Frontiers in Microbiology, 8, 2026. 29089940 10.3389/fmicb.2017.02026 PMC5651101

[fsn31965-bib-0010] Frieri, M. , Kumar, K. , & Boutin, A. (2017). Antimicrobial resistance. Journal of Infection and Public Health, 10(4), 369–378. 27616769 10.1016/j.jiph.2016.08.007

[fsn31965-bib-0011] Henry, R. (2018). Etymologia: *Cronobacter sakazakii* . Emerging Infectious Diseases, 24(11), 2124. 30334728 10.3201/eid2411.180718 PMC6199977

[fsn31965-bib-0012] Holý, O. , Cruz‐Córdova, A. , Xicohtencatl‐Cortes, J. , Hochel, I. , Parra‐Flores, J. , Petrželová, J. , Fačevicová, K. , Forsythe, S. , & Alsonosi, A. (2019). Occurrence of virulence factors in *Cronobacter sakazakii* and *Cronobacter malonaticus* originated from clinical samples. Microbial Pathogenesis, 127, 250–256. 10.1016/j.micpath.2018.12.011 30550840

[fsn31965-bib-0013] Kakatkar, A. , Gautam, R. , Godambe, P. L. , & Shashidhar, R. (2017). Culture dependent and independent studies on emerging food‐borne pathogens *Cronobacter sakazakii*, *Klebsiella pneumoniae* and *Enterococcus faecalis* in Indian food. International Food Research Journal, 24(6), 2645–2651.

[fsn31965-bib-0014] Kilonzo‐Nthenge, A. , Rotich, E. , Godwin, S. , Nahashon, S. , & Chen, F. (2012). Prevalence and antimicrobial resistance of *Cronobacter sakazakii* isolated from domestic kitchens in middle Tennessee, United States. Journal of Food Protection, 75(8), 1512–1517. 10.4315/0362-028X.JFP-11-442 22856579

[fsn31965-bib-0015] Kim, H.‐S. , Kim, Y.‐J. , Chon, J.‐W. , Kim, D.‐H. , Yim, J.‐H. , Kim, H. , & Seo, K.‐H. (2017). Two‐stage label‐free aptasensing platform for rapid detection of *Cronobacter sakazakii* in powdered infant formula. Sensors and Actuators B: Chemical, 239, 94–99. 10.1016/j.snb.2016.07.173

[fsn31965-bib-0016] Li, W. , Raoult, D. , & Fournier, P.‐E. (2009). Bacterial strain typing in the genomic era. FEMS Microbiology Reviews, 33(5), 892–916. 10.1111/j.1574-6976.2009.00182.x 19453749

[fsn31965-bib-0017] Li, Y. , Yu, H. , Jiang, H. , Jiao, Y. , Zhang, Y. , & Shao, J. (2017). Genetic diversity, antimicrobial susceptibility, and biofilm formation of Cronobacter spp. recovered from spices and cereals. Frontiers in Microbiology, 8, 2567. 29312246 10.3389/fmicb.2017.02567 PMC5742210

[fsn31965-bib-0018] Li, Z. , Ge, W. , Li, K. , Gan, J. , Zhang, Y. , Zhang, Q. , & Wang, Q. (2016). Prevalence and characterization of *Cronobacter sakazakii* in retail milk‐based infant and baby foods in Shaanxi. China. Foodborne Pathogens and Disease, 13(4), 221–227. 26886843 10.1089/fpd.2015.2074

[fsn31965-bib-0019] Lönnerdal, B. (2016). Bioactive proteins in human milk: Health, nutrition, and implications for infant formulas. The Journal of Pediatrics, 173, S4–S9. 27234410 10.1016/j.jpeds.2016.02.070

[fsn31965-bib-0020] Losio, M. N. , Pavoni, E. , Finazzi, G. , Agostoni, C. , Daminelli, P. , Dalzini, E. , Varisco, G. , & Cinotti, S. (2018). Preparation of powdered infant formula: Could product's safety be improved? Journal of Pediatric Gastroenterology and Nutrition, 67(4), 543 10.1097/MPG.0000000000002100 30024862 PMC6155361

[fsn31965-bib-0021] Lu, Y. , Liu, P. , Li, C. , Sha, M. , Fang, J. , Gao, J. , Xu, X. , & Matthews, K. R. (2019). Prevalence and genetic diversity of *Cronobacter* species isolated from four infant formula production factories in China. Frontiers in Microbiology, 10, 1938 10.3389/fmicb.2019.01938 31497005 PMC6712172

[fsn31965-bib-0022] Martin, C. R. , Ling, P.‐R. , & Blackburn, G. L. (2016). Review of infant feeding: Key features of breast milk and infant formula. Nutrients, 8(5), 279 10.3390/nu8050279 PMC4882692 27187450

[fsn31965-bib-0023] Mashoufi, A. , Ghazvini, K. , Hashemi, M. , Mobarhan, M. G. , Vakili, V. , & Afshari, A. (2019). A novel primer targeted gyrB gene for the identification of *Cronobacter sakazakii* in powdered infant formulas (PIF) and baby foods in Iran. Journal of Food Safety, 39(2), e12609.

[fsn31965-bib-0024] Mohan Nair, M. K. , Venkitanarayanan, K. , Silbart, L. K. , & Kim, K. S. (2009). Outer membrane protein A (OmpA) of Cronobacter sakazakii binds fibronectin and contributes to invasion of human brain microvascular endothelial cells. Foodborne Pathogens and Disease, 6(4), 495–501. 19415974 10.1089/fpd.2008.0228

[fsn31965-bib-0025] Parra‐Flores, J. , Aguirre, J. , Juneja, V. K. , Jackson, E. E. , Cruz‐Córdova, A. , Silva‐Sanchez, J. , & Forsythe, S. (2018). Virulence and antimicrobial resistance profiles of *Cronobacter sakazakii* and Enterobacter spp. involved in the diarrheic hemorrhagic outbreak in Mexico. Frontiers in Microbiology, 9, 2206. 30319560 10.3389/fmicb.2018.02206 PMC6171480

[fsn31965-bib-0026] Parra‐Flores, J. , Cerda‐Leal, F. , Contreras, A. , Valenzuela‐Riffo, N. , Rodríguez, A. , & Aguirre, J. (2018). *Cronobacter sakazakii* and microbiological parameters in dairy formulas associated with a food alert in Chile. Frontiers in Microbiology, 9, 1–9. 30108565 10.3389/fmicb.2018.01708 PMC6079297

[fsn31965-bib-0027] Pavel, A. B. , & Vasile, C. I. (2012). PyElph‐a software tool for gel images analysis and phylogenetics. BMC Bioinformatics, 13(1), 9 10.1186/1471-2105-13-9 22244131 PMC3299638

[fsn31965-bib-0028] Proudy, I. , Bougle, D. , Coton, E. , Coton, M. , Leclercq, R. , & Vergnaud, M. (2008). Genotypic characterization of Enterobacter sakazakii isolates by PFGE, BOX‐PCR and sequencing of the fliC gene. Journal of Applied Microbiology, 104(1), 26–34. 17850301 10.1111/j.1365-2672.2007.03526.x

[fsn31965-bib-0029] Rohlf, F. J. , & Slice, D. E. (1999). NTSYS‐pc: Numerical taxonomy and multivariate analysis system, version 3.3 Exeter Software. 1, (pp. 30–56). Applied Biostatics Inc.

[fsn31965-bib-0030] Saad, N. M. , & Ewida, R. M. (2018). Incidence of *Cronobacter sakazakii* in dairy‐based desserts. Journal of Advanced Veterinary Research, 8(2), 16–18.

[fsn31965-bib-0031] Singh, N. , Goel, G. , & Raghav, M. (2015). Insights into virulence factors determining the pathogenicity of *Cronobacter sakazakii* . Virulence, 6(5), 433–440. 25950947 10.1080/21505594.2015.1036217 PMC4601314

[fsn31965-bib-0033] Xu, X. , Li, C. , Wu, Q. , Zhang, J. , Huang, J. , & Yang, G. (2015). Prevalence, molecular characterization, and antimicrobial susceptibility of Cronobacter spp. in Chinese ready‐to‐eat foods. International Journal of Food Microbiology, 204, 17–23. 25828706 10.1016/j.ijfoodmicro.2015.03.003

[fsn31965-bib-0034] Yang, B. , Zhao, H. , Cui, S. , Wang, Y. , Xia, X. , Xi, M. , Wang, X. , Meng, J. , & Ge, W. (2014). Prevalence and characterization of *Salmonella enterica* in dried milk‐related infant foods in Shaanxi, China. Journal of Dairy Science, 97(11), 6754–6760. 10.3168/jds.2014-8292 25218754

[fsn31965-bib-0035] Ye, Y. , Wu, Q. , Xu, X. , Yang, X. , Dong, X. , & Zhang, J. (2010). The phenotypic and genotypic characterization of Enterobacter sakazakii strains from infant formula milk. Journal of Dairy Science, 93(6), 2315–2320. 10.3168/jds.2009-2662 20494135

[fsn31965-bib-0036] Zeng, H. , Lei, T. , He, W. , Zhang, J. , Liang, B. , Li, C. , & Wang, J. (2018). Novel multidrug‐resistant Cronobacter sakazakii causing meningitis in neonate, China, 2015. Emerging Infectious Diseases, 24(11), 2121. 30334728 10.3201/eid2411.180718 PMC6199977

[fsn31965-bib-0037] Zeng, H. , Zhang, J. , Li, C. , Xie, T. , Ling, N. , Wu, Q. , & Ye, Y. (2017). The driving force of prophages and CRISPR‐Cas system in the evolution of *Cronobacter sakazakii* . Scientific Reports, 7, 40206 10.1038/srep40206 28057934 PMC5216340

